# Generalized lichen planus pigmentosus and a new management approach

**DOI:** 10.1002/ccr3.8829

**Published:** 2024-05-22

**Authors:** Farnaz Araghi, Farideh Beyki, Azadeh Rakhshan, Hamideh Moravvej Farshi

**Affiliations:** ^1^ Skin Research Center Shahid Beheshti University of Medical Sciences Tehran Iran

**Keywords:** generalized lichen planus pigmentosus, janus kinase inhibitors, lichen planus, tofacitinib

## Abstract

Generalized lichen planus pigmentosus significantly improved with the daily administration of Tofacitinib at a dosage of 15 mg.

## 1 INTRODUCTION

Lichen Planus Pigmentosus (LPP) is an uncommon form of lichen planus (LP), which mostly presents with annular violaceous to brown lesions.

The generalized form of the disease is more prevalent among women in the 3rd to 5th decades of life and the pathogenesis is mostly attributed to hormonal factors.^1^

Skin lesions are mainly distributed over the sun‐exposed areas, including the face and neck. Only a limited number of reports have indicated that the palms and soles could be affected in LPP.^2^ In this study, we are reporting the first case of generalized LPP accompanied by palmar involvement that experienced notable improvement with tofacitinib (Janus Kinase [JAK] inhibitor) after 2 months.

## 2 CASE HISTORY

A 50‐year‐old female patient was referred to our dermatology clinic at Shohadaie‐Tajrish Hospital with widespread skin lesions that had been gradually developing over the past 14 years. The initial manifestation of these lesions was noticed in the groin region. She also experienced mild pruritus. She didn't mention any arthralgia. She only had a history of Hashimoto hypothyroidism, which was effectively managed with levothyroxine. No other medical or drug histories were disclosed.

**FIGURE 1:**
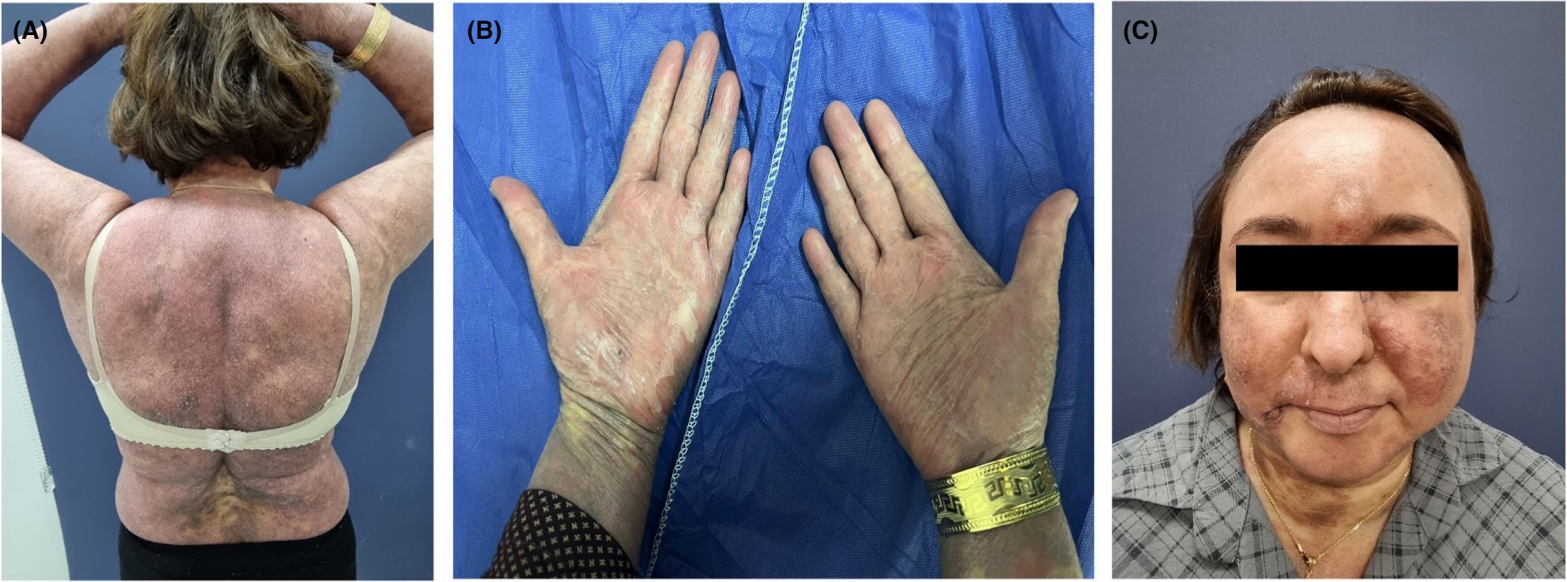
(A) The lesions were mostly violaceous and brown with fine scale, especially on the borders. (B) Exfoliation in the violaceous lesions was detected on the palmar regions of her both hands. (C) Telangiectasia was observed at the center of the lesions predominantly the ones on the face.

In her examination, the lesions were distributed over 90% of her body particularly her face, and other sun‐exposed areas. The lesions were mostly violaceous and brown with fine scale, especially on the borders. Exfoliation in the violaceous lesions was detected on the palmar regions of her both hands. No mucosal involvement has been detected. Furthermore, telangiectasia was observed at the center of the lesions predominantly the ones on the face (Figure 1).

## 3 METHODS

She had undergone two skin biopsies, both of which indicated atypical findings of LPP. Based on this diagnosis, she was prescribed oral prednisolone (20 mg daily); However, after 6 months of treatment, no significant improvements were detected in her lesions. Thus, a subsequent skin biopsy was conducted with differential diagnoses such as Subacute Cutaneous Lupus (SCLE), mixed connective tissue disease, dermatomyositis, systemic lupus erythematosus, LP pigmentosus, LP atrophicus, ashy dermatosis, and Mycosis Fungoides (MF).

**FIGURE 2:**
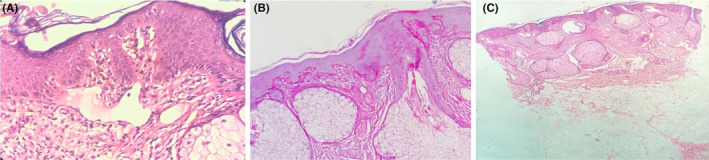
(A) Skin biopsy revealed acanthosis and mild superficial perivascular inflammation (H&E stain, x100). (B) The inflammation produces lichenoid interface reaction with presence of some Civatte bodies in basal layer. Upper dermal melanin incontinence and vascular ectasia are also noted (H&E stain, x400). (C) PAS staining shows mild thickening of basement membrane.

The microscopic examination indicated acanthosis and mild superficial perivascular inflammation. The inflammation produces a lichenoid interface reaction with the presence of some Civatte bodies in the basal layer. Upper dermal melanin incontinence and vascular ectasia are also noted. In addition, PAS staining also revealed mild thickening of the basement membrane (Figure 2). No mucin deposition has been observed by Alcian blue staining. The result of Direct immunofluorescence (DIF) showed multiple globular deposits of immunoglobulin (Ig) G and M at the epidermis and dermoepidermal junction.

Routine laboratory findings showed normal CBC, normal liver enzymes, borderline lipid profile, normal FBS, normal myoglobin, and high LDH (515). Collagen vascular tests such as anti‐SCL‐70, anti‐SSA Ab, anti‐SSB, anti‐Jo‐1 Ab, anti‐smith Ab, and anti‐RNP/Sm IgG were all negative.

A chest Computed Tomography (CT) was performed to rule out any signs of interstitial lung disease.

## 4 CONCLUSION AND RESULT

**FIGURE 3:**
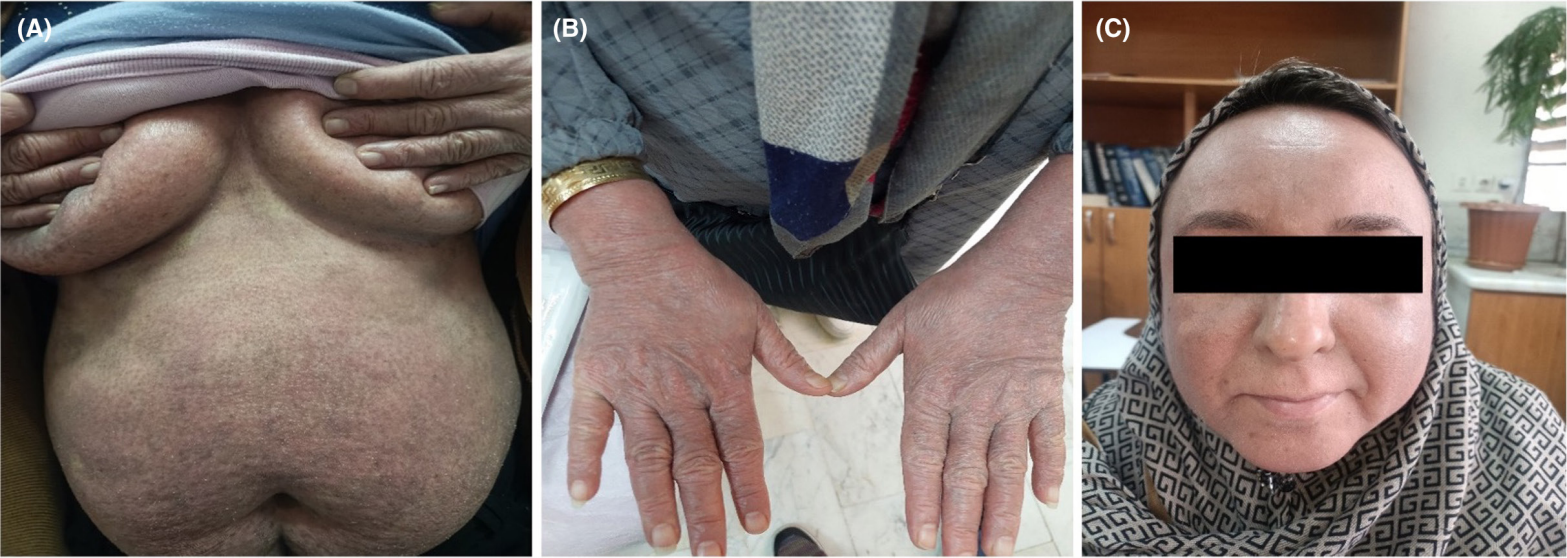
(A, B, C) In the next 4 month's follow‐up, her lesions improved significantly.

Taken together, assuming that the LPP with telangiectasia due to the clinical and histopathological features, Tofacitinib (15 mg daily) was started for the patient. In the next month's follow‐up, her lesions improved significantly (Figure 3). As a result, the medication was continued until the disease was completely resolved.

## 5 DISCUSSION

In this study, we are reporting a case of generalized LPP accompanied by distributed skin lesions over the palmar regions.

It is thought that LPP is an abortive form of LP that a vigorous lichenoid reaction rapidly takes place before the compensatory rise in keratinocyte proliferation, which is commonly observed in typical LP. As a result, there is a rapid transition of papules into brown macules.^3^

Additionally, individuals affected by this condition may also be prone to other autoimmune disorders such as frontal fibrosing alopecia and endocrinopathies like Hashimoto's disease, similar to the case of our patient.^1^

Moreover, according to the preceding records, there exists a variety of eruption patterns for LPP, including diffuse, reticular, blotchy, linear, and perifollicular distributions.^4^ Diffuse form of the disease is a rare variant in which nails and palmoplantar area are often spared;^4^ however, palmar involvement has been observed in a few cases similar to our patient.^2^

In 2018, Dabas et al. conducted a retrospective study of the 10 LPP cases focused on palmoplantar involvement. Like our report, most of the cases experienced rapidly advancing type of LPP, that necessitated a systemic treatment approach.^2^ In this study, all patients experienced the development of palmoplantar lesions after the involvement of other areas of the body similar to our patient.^2^ In addition, the palmar involvement was mostly accompanied by the atypical form of the LPP like our patient.

The inflammation and vulnerability of keratinocytes to destruction are mainly influenced by the signaling of interferon‐g through the JAK—signal transducer and activator of the transcription pathway. In this process, chemokines induced by interferon‐g (such as CXCL9, CXCL10, and CXC11) play a significant role in attracting inflammatory cells to infiltrate the interface dermatitis. Hence, promising therapeutic candidates for addressing LPP are JAK inhibitors.^5^ A recent study highlighted the effectiveness of the topical ruxolutinib on the recalcitrant LPP lesion^5^; however, there is a lack of research on the impact of tofacitinib on the more widespread form of LPP. To our knowledge, this is the first report in which tofacitinib dramatically improves the generalized LPP with palmar involvement following 4 months. Further research is advised to thoroughly examine the impact of tofacitinib on severe cases of LPP in the coming years.

## AUTHOR CONTRIBUTIONS

**Farnaz Araghi:** Data curation; writing – original draft; writing – review and editing. **Farideh Beyki:** Conceptualization; data curation; formal analysis; investigation; project administration. **Azadeh Rakhshan:** Conceptualization; data curation; investigation; project administration. **Hamideh Moravvej Farshi:** Conceptualization; project administration; supervision; writing – review and editing.

## FUNDING INFORMATION

None.

## CONFLICT OF INTEREST STATEMENT

None.

## ETHICS STATEMENT

The ethical issues were completely considered to prepare this case report according to our institution's ethical board guidelines. Moreover, this article was prepared regarding the declaration of Helsinki.

## CONSENT

The patients in this manuscript have given written informed consent to publication of their case details.

## Data Availability

Data openly available in a public repository that issues datasets with DOIs.
